# Risk factors for treatment-related sensorineural hearing loss and hearing aid use in medulloblastoma patients: an observational cohort study

**DOI:** 10.1007/s00066-024-02308-5

**Published:** 2024-10-25

**Authors:** Fabian M. Troschel, David Rene Steike, Julian Roers, Christopher Kittel, Jan Siats, Ross Parfitt, Amélie E. Hesping, Antoinette am Zehnhoff-Dinnesen, Katrin Neumann, Hans Theodor Eich, Sergiu Scobioala

**Affiliations:** 1 Department of Radiation Oncology, Münster University Hospital, Albert-Schweitzer-Campus 1, Building A1, 48149 Münster, Germany; 2 Department of Phoniatrics and Pediatric Audiology, Münster University Hospital, Münster, Germany

**Keywords:** Craniospinal irradiation, Radiotherapy, Cochlea, Chemotherapy, Pediatric cancer

## Abstract

**Purpose:**

This study aimed to analyze treatment-related risk factors for sensorineural hearing loss (SNHL) and an indication for hearing aids (IHA) in medulloblastoma patients after craniospinal radiotherapy (CSRT) and platin-based chemotherapy (PCth).

**Methods:**

A total of 58 patients (116 ears) with medulloblastoma and clinically non-relevant pre-treatment hearing thresholds were included. Cranial radiotherapy and PCth were applied sequentially according to the HIT 2000 study protocol or post-study recommendations, the NOA-07 protocol, or the PNET (primitive neuroectodermal tumor) 5 MB therapy protocol. Audiological outcomes up to a maximum post-therapeutic follow-up of 4 years were assessed. The incidence, post-treatment progression, and time-to-onset of SNHL, defined as Muenster classification grade ≥MS2b, were evaluated. Risk factors for IHA were analyzed separately.

**Results:**

While 39 patients received conventionally fractionated RT (CFRT; group 1), 19 patients received hyperfractionated RT (HFRT; group 2). Over a median follow-up of 40 months, 69.2% of ears in group 1 experienced SNHL ≥MS2b compared to 89.5% in group 2 (*p* = 0.017). In multivariable Cox regressions analysis, younger age and increased mean cochlear radiation dose calculated as the equivalent dose in 2‑Gy fractions (EQD2) were associated with time-to-onset of SNHL ≥MS2b (*p* = 0.019 and *p* = 0.023, respectively) and IHA (*p* < 0.001 and *p* = 0.016, respectively). Tomotherapy and supine positioning were associated with a lower risk for IHA in univariable modelling only (*p* = 0.048 and *p* = 0.027, respectively).

**Conclusion:**

Young age and cochlear EQD2 D_mean_ ≥40 Gy are significant risk factors for the incidence, degree, and time-to-event of SNHL as well as for IHA in medulloblastoma patients.

**Supplementary Information:**

The online version of this article (10.1007/s00066-024-02308-5) contains supplementary material, which is available to authorized users.

## Introduction

Current multimodal treatment methods for medulloblastoma include resection of the primary tumor followed by craniospinal radiotherapy (CSRT) and platin-based chemotherapy (PCth) [1–5]. One possible side effect of this treatment is sensorineural hearing loss (SNHL) resulting from the synergistic ototoxic effect of RT and cisplatin [3, 6–9]. In fact, cranial RT alone may induce hearing loss in many malignancies [10–12]. Here, RT-associated parameters such as radiation technique and cochlear radiation dose (D_mean_ and D_max_) were found to be independent risk factors [4, 13–16]. The application of cisplatin alone is also known to induce relevant ototoxicity, mainly high-frequency SNHL [17–19].

Most studies evaluating treatment-induced SNHL in pediatric patients are constrained by the large variation in irradiation and Cth doses [2, 6, 9, 14, 20]. Conversely, our previous exploratory study in 29 medulloblastoma patients was limited by low patient numbers and short follow-up (less than 2 years) [4].

**Fig. 1:**
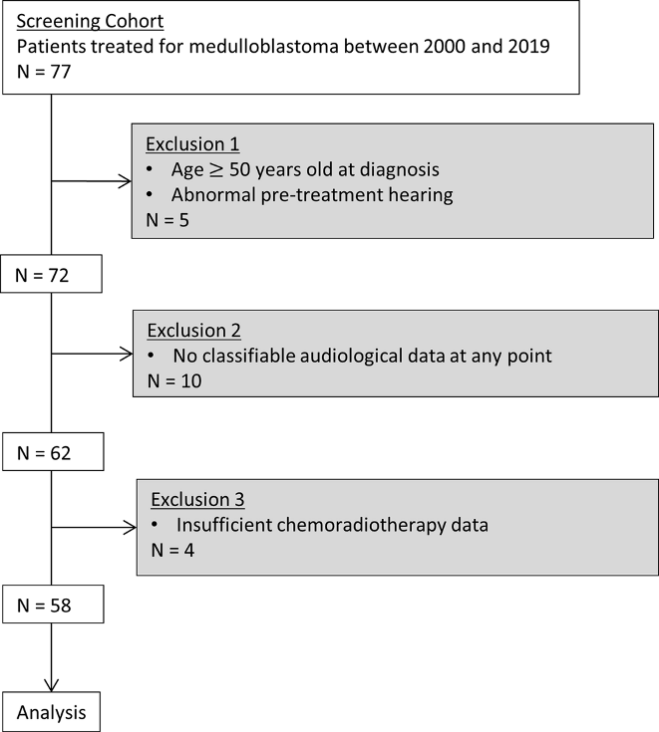
Inclusion and exclusion criteria for the cohort

Herein, we analyze the audiological outcome in an expanded cohort of 58 medulloblastoma patients homogeneously treated according to the HIT 2000 study protocol or the subsequent post-study treatment recommendations (details available online from the German Society for Paediatric Oncology and Haematology (GPOH) website [21]), according to the NOA-07 protocol as available in [22], or according to the PNET (primitive neuroectodermal tumor) 5 MB protocol (details available online from the GPOH website [23] and subsequent publications [24]; all treatment paradigms are summarized in Fig. 1 Supplementary Data). We aimed for a detailed analysis of the impact of radiotherapy parameters including fraction dose, radiation technique, cochlear radiation dose (D_mean_ and D_max_), and radiation position as well as the total cisplatin dose on the incidence, degree, and time-to-onset of hearing impairment as well as on the hearing aid indication (IHA).

## Methods

### Patients

A total of 77 patients with medulloblastoma undergoing treatment between 2000 and 2019 were screened. These patients had diagnosed primary, localized (standard-risk), or metastatic (high-risk) medulloblastoma. Of them, 58 patients with no evidence of hearing loss (HL) prior to the start of their treatment and follow-up audiograms for at least 12 months post-radiation were included in the final cohort (Fig. 1). Key exclusion criteria were no post-treatment audiogram, HL pre-treatment as evidenced by pre-treatment audiograms, and treatment with otoprotective drugs. Full exclusion criteria are described in detail in the Supplementary Data. Patient demographics, treatment variables, and the highest degree of hearing loss during follow-up are presented in Table 1. Patients were mostly treated according to the HIT 2000 study protocol or treatment recommendations, with a minority of patients (some adult patients) treated according to NOA-07 or PNET 5 MB.

**Table 1:** Patient and treatment characteristics as well as hearing outcomes in relation to conventionally fractionated radiotherapy (CFRT) and hyperfractionated radiotherapy (HFRT)

Patient characteristics		All (*n* = 58)	CFRT (*n* = 39)	HFRT (*n* = 19)	*p*-values
Sex (male/female)	Male, *n* (%)	34 (58.6)	21 (53.9)	13 (68.4)	0.29
	Female, *n* (%)	24 (41.4)	18 (46.2)	6 (31.6)	
Age at radiotherapy, years, median (range)		9 (2–36)	9 (2–36)	8 (5–14)	0.80
Age at radiotherapy dichotomized: < 9 years/≥ 9 years	<9 years, *n* (%)	28 (48.3)	10 (46.2)	10 (52.6)	0.64
	≥9 years, *n* (%)	30 (51.7)	21 (53.9)	9 (47.4)	
Median cochlear dose, Gy, median (range; *n* = 57)	Right, median (range)	45.2 (23.9–61.6)	41.5 (23.9–60.6)	48.2 (41.8–61.6)	<0.001*
	Left, median (range)	44.8 (20.5–60.3)	41.9 (20.5–60.3)	48.1 (42.4–59.6)	<0.001*
Median cochlear EQD2 dose, Gy, median (range; *n* = 57)	Right, median (range)	37.7 (22.7–57.6)	39.2 (22.7–57.6)	36.2 (31.4–46.2)	0.36
	Left, median (range)	38.0 (19.5–57.3)	39.6 (19.5–57.3)	36.1 (31.8–44.7)	0.37
Right median cochlear dose dichotomized: < 45 Gy/≥ 45 Gy (*n* = 57)	<45 Gy, *n* (%)	28 (49.1)	26 (68.4)	2 (10.5)	<0.001*
	≥45 Gy, *n* (%)	29 (50.9)	12 (31.6)	17 (89.5)	
Right median EQD2 cochlear dose dichotomized: < 40 Gy/≥ 40 Gy (*n* = 57)	<40 Gy, *n* (%)	36 (62.1)	24 (61.5)	12 (63.2)	0.91
	≥40 Gy, *n* (%)	22 (37.9)	15 (38.5)	7 (36.8)	
Left median cochlear dose dichotomized: < 45 Gy/≥ 45 Gy (*n* = 57)	<45 Gy, *n* (%)	29 (50.9)	24 (63.2)	5 (26.3)	0.009*
	≥45 Gy, *n* (%)	28 (49.1)	14 (36.8)	14 (73.7)	
Left median EQD2 cochlear dose dichotomized: < 40 Gy/≥ 40 Gy (*n* = 57)	<40 Gy, *n* (%)	37 (63.8)	23 (59.0)	14 (73.7)	0.27
	≥40 Gy, *n* (%)	21 (36.2)	16 (41.0)	5 (26.3)	
Maximal cochlear dose, Gy, median (range; *n* = 57)	Right, median (range)	55.2 (29.5–69.7)	53.2 (29.5–61.3)	59.8 (55.9–69.7)	<0.001*
	Left, median (range)	55.4 (30.2–66.4)	52.3 (30.2–65.8)	58.2 (54.8–66.4)	<0.001*
Maximal cochlear EQD2 dose, Gy, median (range; *n* = 57)	Right, median (range)	47.8 (28.0–58.2)	48.9 (28.0–58.2)	44.9 (41.9–52.3)	0.005*
	Left, median (range)	46.5 (28.7–62.5)	49.3 (28.7–62.5)	43.7 (41.1–49.8)	0.004*
Right maximal cochlear dose dichotomized: < 55 Gy/≥ 55 Gy, Gy (*n* = 57)	< 55 Gy, *n* (%)	27 (47.4)	27 (71.1)	0 (0)	<0.001*
	≥55 Gy, *n* (%)	30 (52.6)	11 (29.0)	19 (100)	
Right maximal EQD2 cochlear dose dichotomized: < 40 Gy/≥ 40 Gy (*n* = 57)	<50 Gy, *n* (%)	41 (70.7)	23 (59.0)	18 (94.7)	0.005*
	≥50 Gy, *n* (%)	17 (29.3)	16 (41.0)	1 (5.3)	
Left maximal cochlear dose dichotomized: < 55 Gy/≥ 55 Gy, Gy (*n* = 57)	<55 Gy, *n* (%)	27 (47.4)	25 (65.8)	2 (10.5)	<0.001*
	≥55 Gy, *n* (%)	30 (52.6)	13 (34.2)	17 (89.5)	
Left maximal EQD2 cochlear dose dichotomized: < 40 Gy/≥ 40 Gy (*n* = 57)	<50 Gy, *n* (%)	41 (70.7)	22 (56.4)	19 (100)	0.001*
	≥50 Gy, *n* (%)	17 (29.3)	17 (43.6)	0 (0)	
Total cisplatin dose, mg/m^2^, median (range)		490 (210–560)	560 (280–560)	280 (210–350)	<0.001*
Total cisplatin dose	<300 mg/m^2^, *n* (%)	20 (34.5)	2 (5.1)	18 (94.7)	<0.001*
	≥300 mg/m^2^, *n* (%)	38 (65.5)	37 (94.9)	1 (5.3)	
Total carboplatin dose, mg/m^2^, median (range)		0 (0–2400)	0 (0–1600)	1800 (900–2400)	<0.001*
Radiation technique	2D/3D + IMRT^#^, *n* (%)	32 (55.2)	22 (56.4)	10 (52.6)	0.79
	Tomotherapy, *n* (%)	26 (44.8)	17 (43.6)	9 (47.4)	
Radiation position	Prone, *n* (%)	28 (48.3)	17 (43.6)	11 (57.9)	0.31
	Supine, *n* (%)	30 (51.7)	22 (56.4)	8 (42.1)	
Resection of primary tumor	Total, *n* (%)	33 (56.9)	26 (66.7)	7 (36.8)	0.031*
	Partial, *n* (%)	25 (43.1)	13 (33.3)	12 (63.2)	
M status	M0, *n* (%)	30 (51.7)	26 (66.7)	4 (21.1)	<0.001*
	M+, *n* (%)	28 (48.3)	13 (33.3)	15 (79.0)	
Treatment protocol	HIT-2000, *n* (%)	53 (91.4)	34 (87.2)	19 (100.0)	0.26
	NOA-07, *n* (%)	3 (5.2)	3 (7.7)	0 (0.0)	
	PNET, *n* (%)	2 (3.5)	2 (5.1)	0 (0.0)	
Patients with HL ≥ Muenster 2b, *n* (%)		52 (89.7)	33 (84.6)	19 (100.0)	0.071
Year of treatment dichotomized before 2010/after 2010	Before 2010, *n* (%)	23 (39.7)	14 (35.9)	9 (47.4)	0.40
	After 2010, *n* (%)	35 (60.3)	25 (64.1)	10 (52.6)	
Hearing device use, *n* (%)		19 (32.8)	12 (30.8)	7 (36.8)	0.64
Degree of worst audiological results during follow-up according to the Muenster classification scale, *n* (%) [18]	0	0 (0)	0 (0)	0 (0)	0.21
	1	1 (1.7)	1 (2.6)	0 (0)	
	2a	5 (8.6)	5 (12.8)	0 (0)	
	2b	19 (32.8)	12 (30.8)	7 (36.8)	
	2c	7 (12.1)	6 (15.4)	1 (5.3)	
	3a	10 (17.2)	6 (15.4)	4 (21.1)	
	3b	6 (10.3)	5 (12.8)	1 (5.3)	
	3c	10 (17.2)	4 (10.3)	6 (31.6)	

Radiation doses are given as applied doses and, after balancing, as equivalent dose in 2‑Gy fractions (EQD2) to present biologically equivalent doses

*Statistically significant *p*-value

Each ear of the 58 patients was treated individually, so audiological data of 116 ears were analyzed. The effect of fraction dose, radiation technique, cochlear radiation dose (D_mean_/D_max_), and radiation position as well as total cisplatin dose on development of SNHL and IHA was evaluated (see detailed description in the Supplementary Data). Notably, we collected factually applied chemotherapy doses individually for each patient and did not rely on protocol requirements.

### Audiological methodology

All patients underwent baseline audiometry before RT and post-radiation audiometry usually took place every 3–4 months for the first year, every 5–6 months for the second year, and every 12 months thereafter up to 48 months. Time-to-onset analyses were calculated from the completion of radiotherapy.

Audiological results were grouped as clinically relevant HL (≥ 2b Muenster classification or > 40 dB HL at 4 kHz or above) or clinically non-relevant/normal hearing (Muenster < 2b) [25]. A hearing loss of this degree may be roughly equivalent to difficulty in understanding conversational speech in a quiet environment [26] (although real-world hearing ability also depends on cognitive, intellectual, and language skills; arousal levels; hearing environment; extent (duration and quality) of experience with hearing loss; and social support besides hearing thresholds). This threshold (≥ 2b Muenster classification) was chosen as an increasing body of evidence suggests that the >3 kHz frequency range plays an important role in speech perception [27, 28] and that treatment with hearing aids above 4 kHz is beneficial for many [29, 30]. Thresholds for right and left ears were examined separately for each patient. A detailed description of the audiological methodology is given in the Supplementary Data.

### Radiotherapy

Postoperative RT was applied using either conventional fractionation (CFRT) with five daily fractions per week or hyperfractionated treatment (HFRT) with two daily fractions and ten fractions per week. Treatment techniques included intensity-modulated radiotherapy (IMRT) via tomotherapy or three-dimensional conformal radiotherapy (3D CRT) with dorsoventral static fields for craniospinal axis irradiation (CSI) and an IMRT boost using a sliding-window technique on the posterior cranial fossa and the residual tumor (where required). Cochlear volumes were contoured by resident radiation oncologists and modified by a board-certified radiation oncologist with extensive experience in pediatric radiation oncology. Contouring adhered to European Organisation for Research and Treatment of Cancer (EORTC) guidelines [31]. A visualization of contouring is shown in Supplementary Fig. 2.

Doses were calculated as nominally applied doses and then balanced to reflect biologic equivalency using the equivalent dose in 2‑Gy fractions (EQD2) calculation with an alpha/beta value of 2, similar to previous studies in the field [32–34].

A detailed description of the radiotherapy regimes and techniques is presented in the Supplementary Data.

### Chemotherapy

Chemotherapy recommendations are described in detail in the relevant treatment schemes, i.e., HIT 2000 (updated 2008) and SIOP PNET 5 MB (Supplementary Data, Figures 1a, b, c). According to the HIT treatment recommendations, patients with standard-risk disease receive a total cisplatin dose of 560 mg/m^2^ after CFRT. A total cisplatin dose of 280 mg/m^2^ was administered after HFRT in patients with high-risk disease. PNET 5 MB and NOA-07 treatment protocols were defined for patients with low- or standard-risk disease and a cumulative cisplatin dose of up to 280 mg/m^2^ (PNET 5 MB) or 560 mg/m^2^ (NOA-07) was administered.

### Statistical analyses

Patient and treatment characteristics were assessed using descriptive statistics. Group-based comparisons between characteristics were performed using Mann–Whitney U tests for continuous non-normally distributed parameters and chi^2^ tests were employed to evaluate categorical variables.

Two outcomes were assessed:

- Time-to-onset analyses for HL classified as ≥ 2b according to the Muenster classification. Analyses were performed independently for each ear, as both treatment characteristics (e.g., dose distribution) and outcome measurements (e.g., HL) may reveal side-differing results. Time-to-onset analyses were performed as Kaplan–Meier plots and log-rank tests for bivariate outcomes. Continuous and some bivariate outcomes were also characterized using univariable Cox proportional hazard testing. Statistically significant parameters were then included in a multivariable Cox proportional hazard model, except for those showing strong associations with other covariables.
- Occurrence of a post-therapeutic indication for hearing aid(s). Here, univariable logistic regressions were performed to assess the relationship between individual parameters and a hearing aid indication. Again, statistically significant parameters were then included in a multivariable logistic regression model.

The threshold for statistical significance was defined at *p* < 0.05. Statistical analyses were performed using STATA software version 13.0 (StataCorp LLC, College Station, TX, USA).

Retrospective anonymized single-center data collection of clinical standard-of-care was performed in accordance with the ethical standards of the institutional committee of the University Hospital of Münster (ref. 2014-619). No funding was received for this study.

## Results

### Patient and treatment characteristics

We included 58 patients (34 female and 24 male), most of whom were younger than 10 years old at the time of radiation treatment (median age 9 years, range 2–36 years; Table 1). All patients were treated between 2000 and 2019 and underwent resection and chemoradiotherapy for their disease. In 33 cases (56.9%), the tumor was fully resected while the remainder underwent a partial resection. All patients received subsequent chemotherapy with cisplatin, and some patients were additionally treated with carboplatin. 56/58 patients (96.6%) were treated with CSRT. A total of 8 patients within the cohort were younger than 4 years old when undergoing radiotherapy. Both patients not undergoing CSRT but rather local radiotherapy were part of this group. While CSRT is not recommended for all patients in this subgroup (Supplementary Fig. 1c), all radiotherapy-treated patients in this age group within our cohort showed signs of residual disease after resection or were found to have metastases on imaging or in the cerebrospinal fluid. As is typically recommended in these cases, these patients first underwent systemic therapy after surgery and none of these patients began radiotherapy within 90 days after surgery, while a majority of the remaining patients did.

All patients underwent CFRT (*n* = 39) or HFRT (*n* = 19). 26 patients (44.8%) were treated solely with IMRT via tomotherapy, while the remainder were treated with 3D CRT. Treatment with IMRT showed a high overlap with supine positioning (25/30 cases of supine positioning were IMRT plans, 83.3%), while 3D CRT showed similar associations with prone positioning (27/28 cases of prone positioning were 3D CRT plans, 96.4%; *p* < 0.001).

Median nominal right and left mean cochlear doses were 45.2 and 44.8 Gy, respectively, or 37.7 and 38.0 Gy in EQD2 calculations. In total, 57 ears (50%) received a median dose higher than 45 Gy. 43 ears (37.1%) received an EQD2 dose higher than 40 Gy. Maximal right and left cochlear doses were 55.2 and 55.4 Gy, respectively, or 47.8 and 46.5 Gy in EQD2 calculations. 92 ears (80.7%) received a maximum radiation dose higher than 50 Gy. However, only 34 ears (29.3%) received a maximal EQD2 dose higher than 50 Gy. There was no difference in EQD2 dose to the cochlea in M+ medulloblastoma patients and M0 medulloblastoma patients (median 38.5 vs. 37.7 Gy; *p* = 0.23). Additionally, there was only a very weak correlation between EQD2 mean cochlear dose and patient age, which did not reach the level of significance (Spearman’s rho −0.166; *p* = 0.08).

Overall, 88 ears (75.9%) showed a post-treatment HL of ≥ 2b according to the Muenster classification during follow-up. HL occurred more often in ears from patients treated with HFRT compared to ears from patients treated with CFRT (69.2% vs. 89.5%; *p* = 0.017). After treatment, 19 patients (32.8%) were supplied with hearing devices. Conventional hearing devices were able to fully compensate for post-therapeutic hearing losses. The patients reported did not develop hearing losses of sufficient severity to require cochlear implants (indication for a cochlear implant begins when monaural monosyllabic word comprehension in the free sound field is ≤ 60% at a speech level of 65 dB SPL [35–37] or, correspondingly, if the hearing threshold is above 70 dB HL (over 2–4 frequencies between 0.5 and 4 kHz) [38]).

Patients undergoing HFRT received higher nominal mean and maximal radiation doses to the cochleae (*p* < 0.001), but there was no difference between mean cochlear doses after the EQD2 calculations (*p* = 0.36 and *p* = 0.37). Conversely, the maximum EQD2 dose was slightly higher in conventionally fractionated radiation courses compared to HFRT (48.9 Gy vs. 44.9 Gy, *p* = 0.005 for right and 49.3 Gy vs. 43.7 Gy, *p* = 0.004 for left ears). HFRT-treated patients received lower cisplatin doses (*p* < 0.001) but received more carboplatin treatment (*p* < 0.001). Patients undergoing partial resection were more likely to be treated with HFRT compared to patients with complete resections (*p* = 0.031), as were patients with metastatic disease compared to those without (*p* < 0.001).

### Associations between treatment characteristics and post-treatment hearing loss

**Fig. 2:**
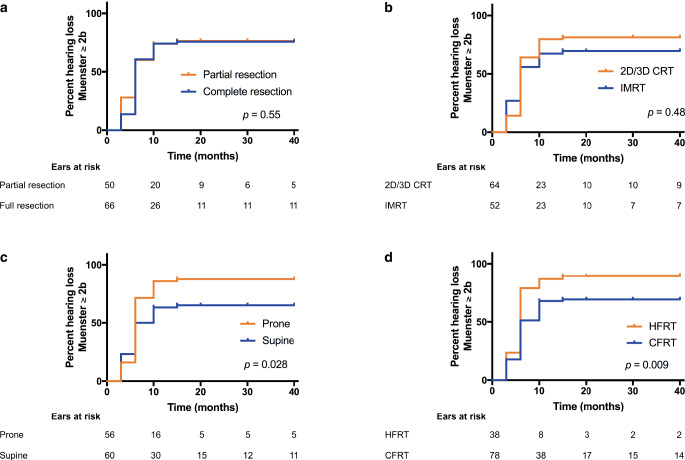
Time-to-onset analysis showing the cumulative percentage of patients with hearing loss ≥ 2b (Muenster classification) over time following the completion of radiation treatment in relation to the extent of tumor resection (**a**), radiation technique (**b**), radiation positioning (**c**), and fraction dose (**d**). Calculations were performed individually for each ear, and “numbers at risk” are given below the graphs. *2D/3D CRT* 2-dimensional/3-dimensional conventional radiotherapy, *IMRT* intensity-modulated radiation therapy, *CFRT* conventionally fractionated radiotherapy, *HFRT* hyperfractionated radiotherapy

**Fig. 3:**
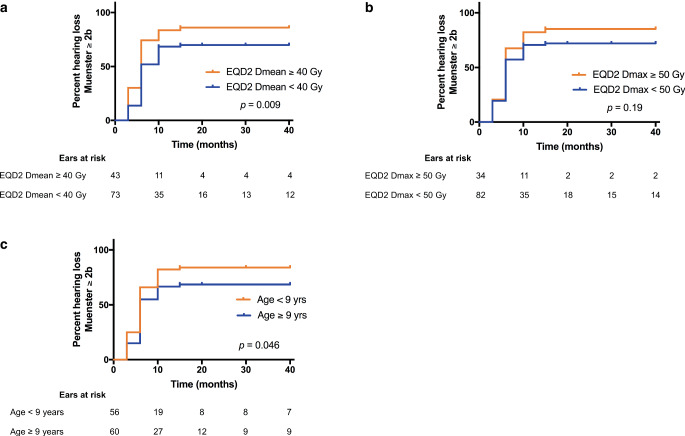
Time-to-onset analysis showing the cumulative percentage of patients with hearing loss ≥ 2b (Muenster classification) over time following the completion of radiation treatment in relation to cochlear mean radiation dose (D_mean_) according to equivalent dose in 2‑Gy fraction (EQD2) calculations (**a**), cochlear maximal radiation dose (D_max_) according to EQD2 calculations (**b**), and age (**c**). Calculations were performed individually for each ear, and “numbers at risk” are given below the graphs

In Kaplan–Meier analyses for the onset of HL ≥ 2b according to the Muenster classification, the extent of resection (complete vs. incomplete) and radiation treatment technique (3D CRT vs. IMRT) did not show associations with HL. However, ears in patients treated with HFRT were more likely to develop ototoxicity compared to those in patients treated with CFRT (*p* = 0.009 in log-rank testing). Similarly, radiation treatment in supine positioning resulted in improved hearing outcomes (*p* = 0.028; Fig. 2). Additionally, an EQD2 D_mean_ higher than 40 Gy (*p* = 0.009) or an EQD2 D_max_ ≥50 Gy (*p* = 0.19) to the cochlea resulted in higher and accelerated HL (Fig. 3a, b). Finally, young age <9 years was also a risk factor for accelerated HL (*p* = 0.046; Fig. 3c).

Median post-therapeutic audiological follow-up time was 40 months. In those diagnosed with HL Muenster ≥ 2b, time-to-onset was 6 months (range 3–15 months). Median time-to-onset was not different between patients treated with HFRT and CFRT (6 months for both groups). In time-to-onset cox hazard regressions, we similarly found that age at the time of radiotherapy (hazard ratio [HR] 0.96; *p* = 0.003), hyperfractionation vs. conventional fractionation (HR 1.57; *p* = 0.041), and the mean EQD2 dose to the cochlea (HR 1.05; *p* = 0.013) were significantly associated with HL. We then performed multivariable modelling of these parameters. The signal for hyperfractionation was lost in multivariable modeling (HR 1.51; *p* = 0.074), but mean EQD2 cochlear dose (HR 1.05; *p* = 0.019) and age at radiotherapy (HR 0.97; *p* = 0.023) remained significantly associated with audiologic outcome (Table 2).

**Table 2:** Univariable and multivariable analyses to identify factors associated with hearing loss and an indication for hearing aid(s)

	Univariable time-to-event analysis for HL ≥ Muenster 2b		Multivariable time-to-event analysis for HL ≥ Muenster 2b		Univariable logistic regression for indication for hearing aid		Multivariable logistic regression for indication for hearing aid	
	HR (CI)	*p-*value	OR (CI)	*p-*value	HR (CI)	*p-*value	HR (CI)	*p-*value
*Sex*	–	0.40	–	**–**	–	0.36	–	**–**
Male	Ref. 1.00		–	**–**	Ref. 1.00		–	**–**
Female	0.83 (0.54–1.28)		–	**–**	1.44 (0.66–3.15)		–	**–**
*Cumulative dose of cisplatin, mg/m*^*2*^	1.00 (1.00–1.00)	0.39	–	–	1.00 (1.00–1.00)	0.81	–	**–**
*Age at radiotherapy, years*	0.96 (0.94–0.99)	0.003*	0.97 (0.95–0.996)	0.023*	0.81 (0.72–0.90)	<0.001*	0.78 (0.68–0.89)	<0.001*
*Equivalent D*_*mean*_* cochlear EQD2, Gy*	1.05 (1.01–1.09)	0.013*	1.05 (1.01–1.09)	0.019*	1.11 (1.03–1.20)	0.005*	1.12 (1.02–1.23)	0.016*
*Equivalent D*_*max*_* cochlear EQD2, Gy*	1.02 (0.98–1.07)	0.35	–	–	1.10 (0.999–1.20)	0.052	–	–
*Year of treatment*	–	0.21	–	–	–	0.24	–	–
Before 2010	Ref. 1.00		–	–	Ref. 1.00		–	–
After 2010	0.76 (0.50–1.17)		–	–	0.62 (0.28–1.37)		–	–
*Radiation technique*	–	0.58	–	–	–	0.048*	–	–
2D/3D CNRT	Ref. 1.00		–	–	Ref. 1.00		–	–
IMRT	0.89 (0.58–1.36)		–	–	0.44 (0.19–0.99)		–	–
*Radiation position*	–	0.09	–	–	–	0.027*	–	0.16
Prone	Ref. 1.00		–	–	Ref. 1.00		Ref. 1.00	
Supine	0.69 (0.45–1.05)		–	–	0.41 (0.18–0.90)		0.49 (0.18–1-32)	
*Fractionation*	–	0.041*	–	0.074	–	0.51	–	–
Conventional	Ref. 1.00		Ref. 1.00		Ref. 1.00		–	–
Hyperfractionation	1.57 (1.02–2.42)		1.51 (0.96–2.38)		1.31 (0.58–2.97)		–	–
*Resection of primary tumor*	–	0.64	–	–	–	0.52	–	–
Total	Ref. 1.00		–	–	Ref. 1.00		–	–
Partial	1.11 (0.72–1.69)		–	–	1.29 (0.59–2.82)		–	–

Radiation doses are given as applied doses and, after balancing with an alpha/beta value of 2, as the equivalent dose in 2‑Gy fractions (EQD2) to present biologically equivalent doses. Cox regression time-to-event analyses were used to evaluate the onset of hearing loss classified ≥ 2b according to the Muenster classification in *n* = 116 ears from 58 patients, of whom 88 met hearing loss criteria over time. Logistic regressions were used to assess factors associated with indication for hearing aid use in *n* = 116 ears from 58 patients, 19 of whom were supplied with hearing aids

*CNRT* conformal radiation therapy, *IMRT* intensity-modulated radiation therapy, *OR* odds ratio, *CI* confidence interval, *HR* hazard ratio, *Ref.* reference

*Statistically significant *p*-value

### Association between treatment characteristics and post-treatment hearing aid use

We also assessed the association between a hearing aid recommendation and patient and treatment characteristics. We found that younger age at the time of radiotherapy (odds ratio [OR] 0.81; *p* < 0.001), higher cochlear EQD2 D_mean_ (OR 1.11; *p* = 0.005), and higher cochlear EQD2 D_max_ (OR 1.10; *p* = 0.052) were associated with IHA. Conversely, patients treated with IMRT as opposed to 3D CRT (OR 0.44; *p* = 0.048) and those undergoing radiotherapy in supine as opposed to prone positioning (OR 0.41; *p* = 0.027) were less likely to require hearing aids. Notably, the cisplatin dose did not show associations with hearing aid use (*p* = 0.81). In a multivariable model including significant parameters (omitting IMRT for collinearity with patient position [*p* < 0.001 in chi^2^ testing]), age (*p* < 0.001) and EQD2 D_mean_ (*p* = 0.016) remained significantly associated with IHA (Table 2).

## Discussion

Herein, we analyzed the post-therapeutic hearing impairment in 58 medulloblastoma patients treated with RT and platin-based Cth. The following treatment-related parameters were assessed:

### Mean radiation dose to the cochlea (cochlear D_mean_)

Cochlear D_mean_ is a known predictive factor for ototoxicity, demonstrating a linear correlation with the incidence and severity of hearing impairment [3, 8, 14, 39]. Our findings support these results, as the cochlear D_mean_ (calculated as equivalent dose using the EQD2 approach considering different fractionation regimens) was the decisive factor for HL and IHA in univariable and multivariable analyses. Our study profits from the availability of IMRT plans (45%), thus allowing for more precise dose calculations. Our data support an EQD2 D_mean_ ≤40 Gy for the cochlea as a threshold, similar to studies in other entities [40, 41], but radiation may need to be lower still to further improve hearing outcomes. The fact that some mean cochlear EQD2 doses exceeded 50 Gy in our study underscores this challenge. While our D_mean_ values were relatively high, they were well in line with other investigations covering a similar study period [5, 42]. Notably, our study does not contradict suggestions to aim for doses of less than 32 or 35 Gy [43, 44] or, more generally, to reduce the radiation dose to the cochlea as much as possible [44], as has also been suggested for other tumors [45]. Here, modern IMRT techniques and removal of flattening filters may allow for more substantial dose reductions [46]. Proton therapy may also reduce the dose to the cochlea, with some [47, 48] but not all [49] studies finding favorable results for proton therapy. However, even in a comparatively large study describing a dosimetric advantage for proton therapy, no differences were seen for severe clinical ototoxicity, leading to overall conflicting results [48].

### Radiotherapy fractionation

Bhandare et al. reported that the fraction dose had no effect on the incidence of SNHL in patients with head and neck tumors, although the median time until persistent hearing alterations was found to be longer for CFRT than for HFRT [13]. Lannering et al. found no significant difference in post-treatment HL between CFRT- and HFRT-treated patients despite higher doses to the cochlea after HFRT compared to CFRT [5]. The authors hypothesized that HFRT treatment induces a radiosensitizing redistribution of proliferating tumor cells while not similarly affecting normal tissue.

In contrast to these results from Lannering’s trial, our analysis showed that HFRT was associated with inferior hearing outcomes compared to CFRT in univariable analyses, both in terms of incidence and in terms of time-to-event analyses. Notably, the radiation dose to the cochlea was also significantly higher in our study for HFRT vs. CFRT, similar to Lannering’s study [5]. Importantly, once EQD2 standardization was used in our study, no difference was seen in mean doses, indicating no biological difference in treatment intensity (no EQD2 calculations were performed for cochlear doses in [5]). The difference in hearing outcomes between the two studies may have resulted from differences in radiation technique (in our study, more patients were treated with IMRT) and ototoxicity scores (HIT and Brock scores were used by Lammering et al., while we used the Muenster score) [50, 51]. Nonetheless, our study found that hyperfractionation does not shield patients from the ototoxicity associated with higher radiation doses to the cochlea. However, at the same time, hyperfractionation was also not an independent risk factor for higher ototoxicity, as the statistical difference between HFRT and CFRT was lost in multivariable modelling once the mean EQD2 cochlear radiation dose was introduced to the model as a covariable. In summary, HFRT neither protected nor substantially compromised hearing in our study (if anything, it was a marginally negative factor); mean EQD2 cochlear radiation dose—independent of fractionation—seemed to be the main determinant of hearing outcomes in the cohort.

### Radiation technique, treatment year, and radiation positioning

We found a tendency towards a lower risk of SNHL and a significantly lower risk of IHA for tomotherapy compared to 3D CRT. As suggested by Huang et al., IMRT delivers a lower dose per fraction to the cochlea compared to CNRT, with a probable decrease in biologic effect to the organ [16]. Further aspects such as steeper dose gradients from the tumor region to the cochlea and a smaller cochlear volume in IMRT plans, as well as better imaging modalities using the IMRT/IGRT technique, may also improve post-therapeutic hearing outcomes [3, 13, 16, 52–55].

This likely also corresponds with our finding that treatment after 2010 seemed to result in lower treatment-related ototoxicity, as IMRT techniques have only been fully implemented in the past decade.

Unsurprisingly, we found a significant overlap between patients treated with IMRT plans and treatment administration in a supine position. We believe that the advantages of supine treatment for hearing outcomes may be primarily explained via the increased use of modern IMRT techniques in this subgroup.

### Cisplatin

Cisplatin is known to be a key factor for bilateral, irreversible, progressive, high-frequency SNHL [17–19]. Several studies showed an enhanced hearing injury from the synergistic ototoxic effects of cranial RT and cisplatin as well as a direct correlation between HL and the cumulative cisplatin dose [3, 4, 6–8, 56, 57]. Conversely, Lannering et al. found no relation between the incidence and severity of HL and the number of cisplatin courses received in more than 300 patients [5].

In our study, we found no correlation between the total cisplatin dose and audiological outcome. This likely indicates that D_mean_ is the more immediately relevant risk factor for ototoxicity, in line with other studies [13, 15, 18, 43, 58]. Histopathological inner ear findings may explain this hypothesis (see Supplementary Data). However, our study was not powered to fully assess the effects of chemotherapy on hearing outcomes, as the multitude of treatment regimens (cisplatin ± carboplatin, with both applied at individualized doses) would have necessitated subgroup analyses that we were unable to perform considering the limited patient number of the overall cohort. Hence, we cannot make definitive statements regarding the interplay between chemotherapy and audiological outcomes based on our data.

### Age, sex, and resection of primary tumor

The literature regarding the role of age in terms of the post-treatment hearing threshold is inconclusive, as both older [13, 34, 59, 60] and younger age [6, 20, 58] have been associated with ototoxicity. Similarly, sex had no effect in some [4, 13, 18] but a direct relationship with post-treatment SNHL in other studies [59, 61] in pediatric patients. Our findings indicate that young age is a risk factor for SNHL as well as for IHA, in line with a very recent study in a large cohort of childhood cancer survivors [62]. One possible mechanism by which to explain this is the higher risk of developing post-irradiation hyperemia in younger children, which could cause increased vulnerability to cisplatin damage within the cochlea [63]. Cochlear/cranial irradiation, especially in children under 5 years, may damage the integrity of membranous inner ear barriers or central nervous system barriers, thereby reducing the normal inner ear tissue tolerance to cisplatin [64]. Notably, there was no significant correlation between age and dose to the cochlea in our cohort, and age-related effects were independent of the EQD2 D_mean_ values in multivariable modelling.

No systematic effect of sex on audiological outcome was found in our study.

We found no association between the extent of resection of the primary tumor and HL. This is unexpected considering that all patients with residual tumor were treated with HFRT and, consequently, with higher nominal cochlear radiation doses compared to patients with complete resection, who received CFRT. Currently, there are no data in the literature demonstrating a correlation between the extent of medulloblastoma resection and ototoxicity.

### Strengths and limitations

First, medulloblastoma remains a rare disease. While our study was able to draw on a comparatively large sample relative to the rarity of the disease, the cohort was nonetheless too small for extensive multivariable modeling and had to leave some questions unanswered. However, audiological follow-up was relatively common and allowed for stringent analyses. Second, this study may have suffered from selection bias due to its observational nature and follow-up was not universally available. One key advantage was the strict exclusion of patients with pre-existing audiological deficiencies, so that any follow-up hearing loss was reasonably attributable to treatment side effects. Consequently, the chemotherapy dose was not pre-emptively reduced from the start due to any prior hearing loss, making the application and comparability of chemotherapy effects more consistent. However, dose reductions towards the end of chemotherapy cycles in the case of an early onset of hearing loss cannot be discounted. Third, cutoffs for age-based risk assessment are inconsistent in the literature. To circumvent this issue, we consistently performed calculations using age as a continuous variable and simply used the median of our cohort (9 years) for exploratory cutoff analyses. Finally, owing to the long period of patient inclusion, cochlear volume contouring guidelines evolved and, more importantly, CT resolution increased. However, we previously demonstrated that the subsequent decrease in cochlear contouring volume over the study period was non-significant [4] and, hence, no major effects on D_mean_ calculations are likely. Modern recommendations such as those from the hearing loss task force from the Pediatric Normal Tissue Effects in the Clinic (PENTEC) group [62] were not available during our study but may help to reduce ototoxicity in pediatric radiation oncology in the future.

## Conclusion

Treatment-related ototoxicity and hearing aid use is common in medulloblastoma patients after multimodal therapy. Young age and a higher mean cochlear radiation dose were unfavorable for audiological outcomes in our cohort. Independent of the cochlear dose, hyperfractionation did not protect hearing. The IMRT radiation technique showed trends towards improved outcomes. Modern techniques and careful consideration of cochlear radiation dose may reduce long-term ototoxicity in medulloblastoma patients.

## Supplementary Information

Description of additional Methods, including treatment sequence (Supplementary Figure 1), delineation of the cochlea (Supplementary Figure 2), and additional considerations of radiation-induced histopathologic inner ear changes.
