# Supplementary material for: Risk factors for treatment-related sensorineural hearing loss and hearing aid use in medulloblastoma patients: an observational cohort study
Source: Strahlenther Onkol. 2024 Oct 25;201(4):438–51. doi: 10.1007/s00066-024-02308-5 (PMC11928435; doi:10.1007/s00066-024-02308-5)
Supplement: Supplementary file 1 — Description of additional Methods, including treatment sequence (Supplementary Figure 1), delineation of the cochlea (Supplementary Figure 2), and additional considerations of radiation-induced histopathologic inner ear changes. [file 66_2024_2308_MOESM1_ESM.docx]

**Supplementary Material**

**Methods:**

*Patients*

Patients were excluded from the study if:

- they were older than 50 years,
- the pre-treatment audiogram showed thresholds outside of the normal range (20 dB HL),
- they had no available post-treatment audiological data for at least 12 months post-radiation,
- they had no available radiation treatment plans,
- they underwent more than one change between types of PCth treatment,
- platin administration and radiotherapy overlapped, or if
- they were treated with otoprotective drugs, including amifostine or sodium thiosulfate.

*Audiological methodology*

Audiological test methodology adhered to international standards (e.g., ISO 8253-1:1998). Requirements for audiometric test equipment (e.g., IEC 60645-1:2001) and the calibration of that equipment (e.g., ISO 389-1:2000) were similarly met. Audiological tests were conducted in the Department of Phoniatrics and Pediatric Audiology, University Hospital of Muenster (Germany).

Radiotherapy-specific hearing threshold data were used to address research questions on the incidence, degree, progression, and time-to-onset of HL. The stimuli used were pure tones within a frequency range of 0.25-8 kHz or higher where possible. The audiological results were classified according to the Muenster Classification of High Frequency Hearing Loss, an ordinal scale of the severity of ototoxicity [1]. Classification data were grouped into clinically-relevant HL (Muenster ≥ 2b) or clinically-non-relevant/normal hearing (Muenster < 2b) [1, 2]. The key definer of the Muenster 2b classification is the worst hearing threshold being > 40 dB HL at 4 kHz or above, with no thresholds lower than 4 kHz being worse than 20 dB HL.

To avoid incorporating any potential effects of conductive hearing losses (CHL) common in childhood and unrelated to ototoxicity into our study, we used bone-conduction (BC) thresholds for audiogram classification where available in cases of possible CHL. Ambiguities were clarified with tympanometric results where available. Where there remained a lack of clarity regarding low-frequency or pan-tonally raised air-conduction thresholds, these audiograms were not classified. The analysis of post-treatment HL was based on each patient’s worst classified audiogram after the end of platin/CRT treatment. Thresholds for right and left ears were examined separately for each patient.

*Radiation therapy*

The delineation of the gross tumor volume (GTV), clinical target volume (CTV), and planning target volume (PTV) was based on the treatment recommendations issued for the HIT 2000, NOA-07, and the PNET 5MB studies. For all treatment protocols, the radiation volume for craniospinal axis (CSA) covered the entire subarachnoid volume including the cribriform plate and temporal fossae intracranially and the thecal sac caudally (usually at the level of S2/3). Further dose escalation occurred on the entire posterior cranial fossa (including the tumor bed) for patients treated with HIT 2000 or NOA-07 protocols, or on the tumor bed of the primary tumor site for PNET 5MB patients. The GTV for the primary tumor bed was defined based on the T1- and T2-weighted 1.5T magnetic resonance images fused with a thin-cut 3mm computed tomography scan. CTV definition was based on post-chemotherapeutic or postoperative imaging with a safety margin of 5mm. PTV of the primary tumor covered the postoperative tumor bed with safety margins of 10mm and were anatomically confined. Treatment plans were generated in accordance with the International Commission on Radiation Units and Measurements (ICRU) reports 50, 62 and 83, depending on the RT technique employed [3, 4].

Medulloblastoma Therapy recommendations issued for the HIT 2000 study (updated 2008) note that the cumulative dose and radiotherapy fractionation are dependent upon the presence of solid intracerebral and/or spinal metastases (Fig. 1A, Supplemental data). CFRT is recommended for non-metastatic disease or where microscopic metastases are present in the liquor. The cumulative dose for CSI was calculated as 35.2 Gy with 1.6 Gy/fraction. The dose for the PCF and initial tumor bed was escalated to 55 Gy with 1.8 Gy/fraction. HFRT was used primarily for patients with macroscopic metastases (high-risk disease) in accordance with the HIT 2000 guidelines. The original protocol investigated hyperfractionation in non-metastatic patients as well, and a small number of patients in the study were treated according to the protocol, not the (later-published) guidelines. The target dose was 40 Gy for the CSA, 60 Gy for the PCF and 68 Gy for primary tumor bed using 1 Gy/fraction twice a day (**Supplementary Fig. 1A**). An alternative medulloblastoma therapy protocol SIOP PNET 5 MB is destined for patients with a low- or standard-risk medulloblastoma (**Supplementary Fig. 1B**). According to this protocol, CFRT is recommended. Depending on age and risk status, a dose of 23.4 Gy or 36 Gy with 1.8 Gy/fraction was applied to the craniospinal axis. The dose for the PCF was escalated to 54 Gy with 1.8 Gy/fraction. Children younger than 4 years underwent treatment according to the HIT 2000 therapy recommendations for their age group (**Supplementary Figure 1C**). Here, craniospinal irradiation was only suggested in case of metastases.


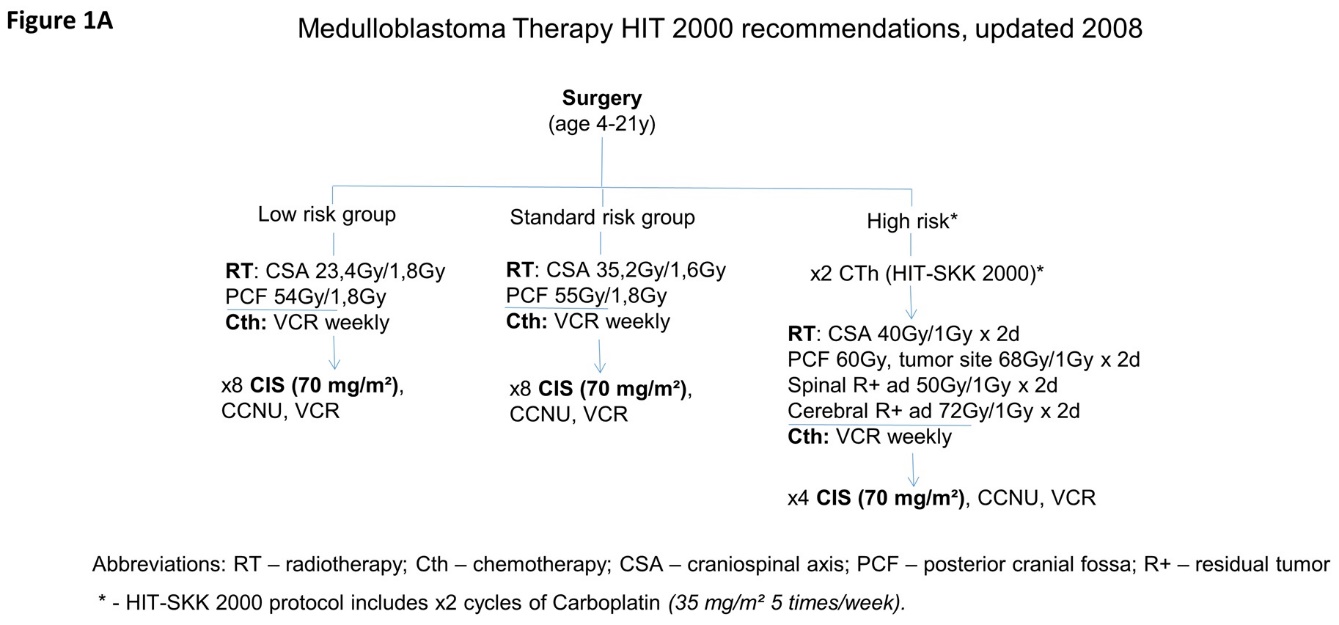


**Supplementary Figure 1A**: **Medulloblastoma Therapy HIT 2000 recommendations, updated 2008**, for children and young adults 4-21 years old.


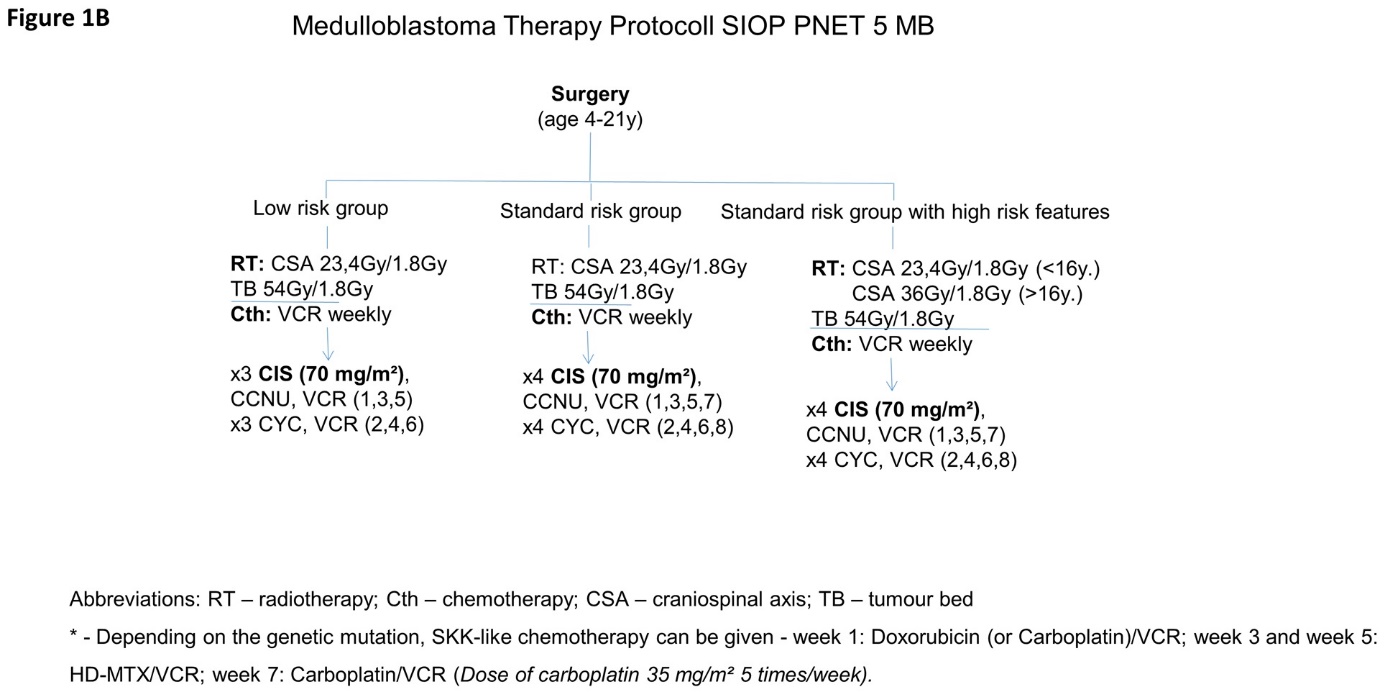


**Supplementary Figure 1B**: **Medulloblastoma Therapy Protocol SIOP PNET 5B**.


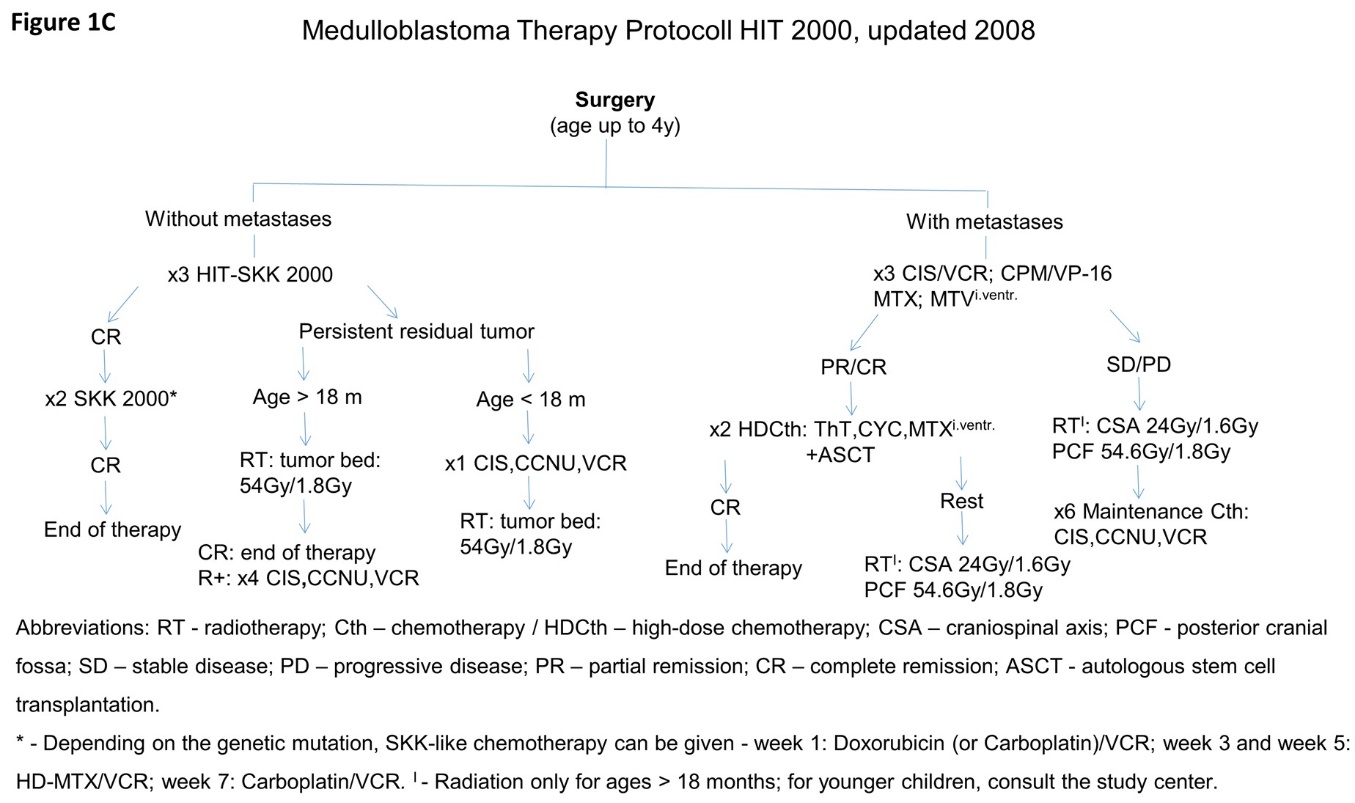


**Supplementary Figure 1C**: **Medulloblastoma Therapy HIT 2000 recommendations, updated 2008**, for children younger than 4 years old.

The radiation fields were adapted to the organ at risk (OAR) in each individual case. The dose-volume objectives met the criteria for OAR outlined in the HIT 2000, NOA-07, or PNET 5 MB protocols as well as in the Quantitative Analyses of Normal Tissue Effects in the Clinic (QUANTEC) reports. The median Dmean and maximal Dmax cochlear radiation doses were assessed separately for each ear. The radiation exposure of the cochlea was presented in differential dose-volume histograms (DVH).

Tomotherapy plans were generated in accordance with ICRU report 83 and assessed using Tomo Planning System Version 5 (Accuray Inc., Sunnyvale, USA). The plans were created for the High Art HDII tomotherapy system that uses a helical slice 6-MV photon beam modulated by 64 binary multileaf collimator leafs. The Co60-system or Siemens linear accelerator PRIMUS (Siemens Medical Solutions, Concord, USA) were used for combined treatment in the earlier years and the Varian True Beam linear accelerator (Varian Medical Systems, Palo Alto USA) was later used for radiation application. CSI was performed in prone positioning using symmetrical bilateral cranial fields and dorso-ventral spine fields. A 7-field-Step and shoot-IMRT was used for the additional dose to the posterior fossa. The treatment was created for a Primus™ LINAC (Siemens, Medical Solutions) with energy of 15 MeV.  Importantly, dose coverage, especially to the posterior fossa, was generally not sacrificed to protect the inner ear. Conversely, we relied on modern radiation delivery techniques to reduce dose to the cochlea (as described above).

Contouring of the cochlea is demonstrated in **Supplementary Figure 2**.


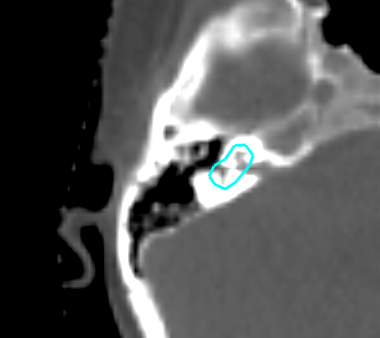


**Supplementary Figure 2.** Cochlear contouring on a radiation planning CT scan. Contouring was performed according to EORTC guidelines [5] and inner ear structures were identified on CT bone window settings.

*Histopathological findings in inner ear structures after CRT and cisplatin treatment*

According to Nader and Gidley, SNHL after radiation could result from damage to the organ of Corti, atrophy of the basilar membrane, spiral ligament, and stria vascularis [6]. A loss of outer hair cells in the basal turn of the cochlea follows oxidative damage caused by increased production of reactive oxygen species (ROS) leading to cell necrosis [7]. The deterioration is progressive over time. Cisplatin ototoxicity is due to the production of ROS that then trigger apoptosis in cochlear hair cells [8]. Nader and Gidley suggest that HL is caused by the loss of inner and outer hair cells, atrophy of the stria vascularis and degeneration of the spiral ganglion and the cochlear nerve [6]. Breglio et al. demonstrated long-term retention of cisplatin in the human cochlea resulting in a higher concentration in the stria vascularis [9].

Progressive radiation-induced vascular fibrosis of vessels supplying the inner ear may also be a factor underlying the role played by ototoxic RT in the deterioration of hearing over the long term [10, 11]. Kovar and Waltner, in a case study, found fibrosis around the obliterated vessels of the modiolus seven months after radiation [10]. Hoistad et al. observed histopathological changes in arterioles three months after and increasing arterioral fibrosis and intimal occlusion 15 months after irradiation [11]. Their histopathological findings after the combined application of cisplatin and radiation did not significantly differ from findings after treatment with cisplatin alone or radiation alone during this observation period. They assumed that chronic damage to the vaso-connective tissue caused by radiation may be the most important factor in post-therapeutic hearing loss with progressive vascular fibrosis and obliteration over a long period of time.

*References*

1. Schmidt CM, Bartholomäus E, Deuster D, Heinecke A, Dinnesen AG (2007) Die “Münsteraner klassifikation”. Eine neue einteilung der hochtonschwerhörigkeit nach cisplatingabe. HNO 55:299–306. https://doi.org/10.1007/s00106-005-1368-1

2. Bhandare N, Jackson A, Eisbruch A, Pan CC, Flickinger JC, Antonelli P, Mendenhall WM (2010) Radiation Therapy and Hearing Loss. Int J Radiat Oncol Biol Phys 76:. https://doi.org/10.1016/j.ijrobp.2009.04.096

3. Landberg T, Chavaudra J, Dobbs J, Gerard J-P, Hanks G, Horiot J-C, Johansson K-A, Möller T, Purdy J, Suntharalingam N, Svensson H (1999) Reports of the International Commission on Radiation Units and Measurements Volume os-32, Issue 1: ICRU Report 62: Prescribing, Recording and Reporting Photon Beam Therapy (Supplement to ICRU Report 50). J ICRU os-32:

4. Hodapp N (2012) The ICRU Report No. 83: Prescribing, recording and reporting photon-beam intensity-modulated radiation therapy (IMRT). Strahlentherapie und Onkol 188:97–99. https://doi.org/10.1007/s00066-011-0015-x

5. Brouwer CL, Steenbakkers RJHM, Bourhis J, Budach W, Grau C, Grégoire V, Van Herk M, Lee A, Maingon P, Nutting C, O’Sullivan B, Porceddu S V., Rosenthal DI, Sijtsema NM, Langendijk JA (2015) CT-based delineation of organs at risk in the head and neck region: DAHANCA, EORTC, GORTEC, HKNPCSG, NCIC CTG, NCRI, NRG Oncology and TROG consensus guidelines. Radiother Oncol 117:83–90. https://doi.org/10.1016/j.radonc.2015.07.041

6. Nader ME, Gidley PW (2019) Challenges of Hearing Rehabilitation after Radiation and Chemotherapy. J Neurol Surgery, Part B Skull Base 80:214–224. https://doi.org/10.1055/s-0039-1677865

7. Low WK, Tan MGK, Sun L, Chua AWC, Goh LK, Wang DY (2006) Dose-dependant radiation-induced apoptosis in a cochlear cell-line. Apoptosis 11:2127–2136. https://doi.org/10.1007/s10495-006-0285-4

8. Kamogashira T, Fujimoto C, Yamasoba T (2015) Reactive oxygen species, apoptosis, and mitochondrial dysfunction in hearing loss. Biomed Res Int 2015:. https://doi.org/10.1155/2015/617207

9. Breglio AM, Rusheen AE, Shide ED, Fernandez KA, Spielbauer KK, McLachlin KM, Hall MD, Amable L, Cunningham LL (2017) Cisplatin is retained in the cochlea indefinitely following chemotherapy. Nat Commun 8:. https://doi.org/10.1038/s41467-017-01837-1

10. Kovar M, Waltner JG (1971) Radiation effect on the middle and inner ear. Pract Otorhinolaryngol (Basel) 33:233–42. https://doi.org/10.1159/000275001

11. Hoistad DL, Ondrey FG, Mutlu C, Schachern PA, Paparella MM, Adams GL (1998) Histopathology of human temporal bone after cis-platinum, radiation, or both. Otolaryngol - Head Neck Surg 118:825–832. https://doi.org/10.1016/S0194-5998(98)70276-1
